# Endovascular treatment of bilateral isolated aneurysm of the internal iliac artery

**DOI:** 10.1590/1677-5449.180115

**Published:** 2019-04-17

**Authors:** Edwaldo Edner Joviliano, Daniela Vieira, Letícia da Silva Moreira, André Luís Foroni Casas

**Affiliations:** 1 Cirurgia Vascular, Universidade de São Paulo – USP, Ribeirão Preto, SP, Brasil.; 2 Cirurgia Vascular, Universidade de Franca – UNIFRAN, Franca, SP, Brasil.

**Keywords:** iliac aneurysm, endovascular procedures, stents

## Abstract

Isolated aneurysms of the iliac arteries comprise less than 2% of abdominal aneurysms. The internal iliac artery is involved in 10-30% of cases. In most cases patients are asymptomatic, unless rupture occurs. They can be diagnosed by Doppler ultrasonography, magnetic resonance imaging or, preferably, angiotomography. Significant expansion, diameter of 3 cm or greater, and symptomatic cases are indications for surgery. We present the case of a patient with an incidental ultrasonographic finding of bilateral aneurysm of the internal iliac arteries, both with indications for surgery. The patient was successfully treated with endovascular techniques, first repairing the right internal iliac with a branched iliac stent graft, preserving patency, then embolizing the left internal iliac artery. Knowledge of the various different techniques and devices and their limitations is fundamental to adequate planning of endovascular treatment, even in rare cases.

## INTRODUCTION

Iliac artery aneurysms (IAA) account for less than 2% of all abdominal aneurysms and affect 0.3-0.6% of the general population.^1^ The internal iliac artery is involved in 10-30% of cases, and the aneurysm is bilateral in half of the cases.^2^^-^ 6 The mortality rate in cases treated electively with open procedures is 10%. Mortality rises to 33-50% when the aneurysm ruptures.^7^^,^ 8 In some cases, this is the initial presentation, making treatment difficult.

The majority of IAA are asymptomatic, making diagnosis less likely,^2^ and the majority remain asymptomatic until they rupture or are diagnosed incidentally.^9^ Rupture occurs in 38-51% of cases and characteristically presents with acute progressive pain, hypotension, and a pulsating mass in the lower abdomen and inguinal areas. Iliac artery aneurysms can occur in the retroperitoneal or intraperitoneal spaces, compressing the rectum, ureter, or bladder.^8^ More recently, the wider availability and greater sensitivity of imaging techniques have led to increases in early diagnosis of these aneurysms.^9^

Noninvasive imaging exams are often part of incidental diagnoses, but the gold standard is helical computed angiotomography.^2^^,^ 3 ^,^ 8

The natural history of internal iliac artery aneurysms is still unclear, but several authors recommend repairing these aneurysms when diameter exceeds 3 cm, since the risk of rupture in such cases is 14-31%. Clinical management is reserved for asymptomatic patients with aneurysms smaller than 3 cm. However, it should be pointed out that there are reports of rupture of aneurysms with diameters of less than 3 cm.^4^ Feasible technical approaches include endovascular and open methods. Nowadays, open surgery is only considered in cases with anatomic variations, because complications and mortality rates are elevated (11 to 33%).^3^ Additionally, internal iliac aneurysms have certain peculiarities that make open approaches difficult (deep location within the pelvis, intimate relationships with veins, proximity to the ureter, and difficulties exposing the distal branches). Currently, endovascular treatment is the first choice, since it is invasive, reduces the risk of intraoperative hemorrhages and renal failure and reduces the duration of hospital stays.^2^^,^ 10 ^,^ 11

While countless endovascular techniques exist, the choice of treatment depends on the anatomy of the injury and the experience of the team. Generally, exclusion of the aneurysm is recommended, since it is associated with fewer complications and better long-term results.^2^^,^ 5 ^,^ 12 ^-^ 19

### Part I – Clinical situation

The patient was a 69-year-old male, with a prior history of pain in the hypogastric area and the left flank. During investigation of these pains, an ultrasound examination of the kidneys and urinary tracts was ordered. This did not detect any abnormalities of the urinary system, but revealed aneurysms of both internal iliac arteries. The patient’s only risk factor was systemic arterial hypertension. There was no family history of aneurysmal disease.

At that time, the patient was in good general health. He stated that he did not have erectile dysfunction and was free from pain. Arterial blood pressure was 130/80 mmHg and heart rate was 70 bpm. Pulmonary and cardiac examinations did not reveal any abnormalities. His abdomen was flat, flaccid, and painless on palpation. Femoral, popliteal, posterior tibial, and dorsal pedal pulses were all palpable and the ankle-brachial index was normal bilaterally. The patient was taking 50 mg losartan potassium orally once a day.

**Figure 1:**
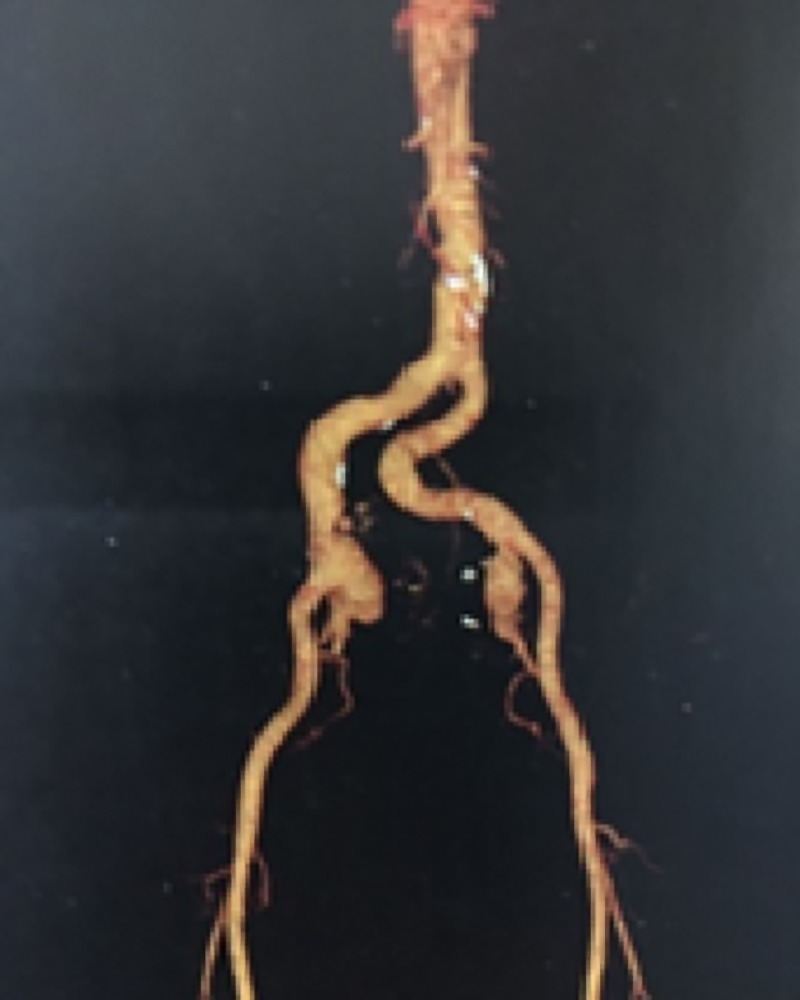
Fusiform aneurysms of the internal iliac arteries.

Angiotomography showed a fusiform aneurysm of the right internal iliac artery, with a maximum diameter of 4.2 cm and length of 5.9 cm, and a fusiform aneurysm of the left internal iliac artery, with a maximum diameter of 3.5 cm and length of 4.7 cm (Figure 1). The diameter of the infrarenal aorta was 20 mm, the right common iliac artery had a diameter of 18 mm, the left common iliac artery diameter was 14 mm, and both external iliac arteries were 11 mm in diameter. The angiotomographic examination of the lower limbs was normal.

The laboratory test results were as follows, hemoglobin: 12.4 g/dL; hematocrit: 36%; platelets: 408,000/mm^3^; glucose: 85 mg/dL; sodium: 142 mEq/mL; potassium: 3.7 mEq/L; creatinine: 1.0 mg/dL; urea: 40 mg/dL; activated partial thromboplastin time (APTT): 33.8 s; international normalized ratio (INR): 1.2; and prothrombin activity: 62%.

A number of treatment options were considered for this presentation:

1 - Bilateral iliac grafting with branched endoprosthesis, preserving both internal iliac arteries;
2 - Unilateral iliac graft with branched endoprosthesis, preserving one internal iliac artery, and embolization of the other;
3 - Embolization of both internal iliac arteries, sequentially;
4 - Aortoiliac endoprosthesis;
5 - Open surgical treatment, with ligature of both aneurysms.

### Part II – What was done

The decision was taken to employ endovascular treatment, with the intention to initially treat the larger aneurysm and preserve the internal iliac artery, treating the smaller aneurysm (embolization) later.

**Figure 2:**
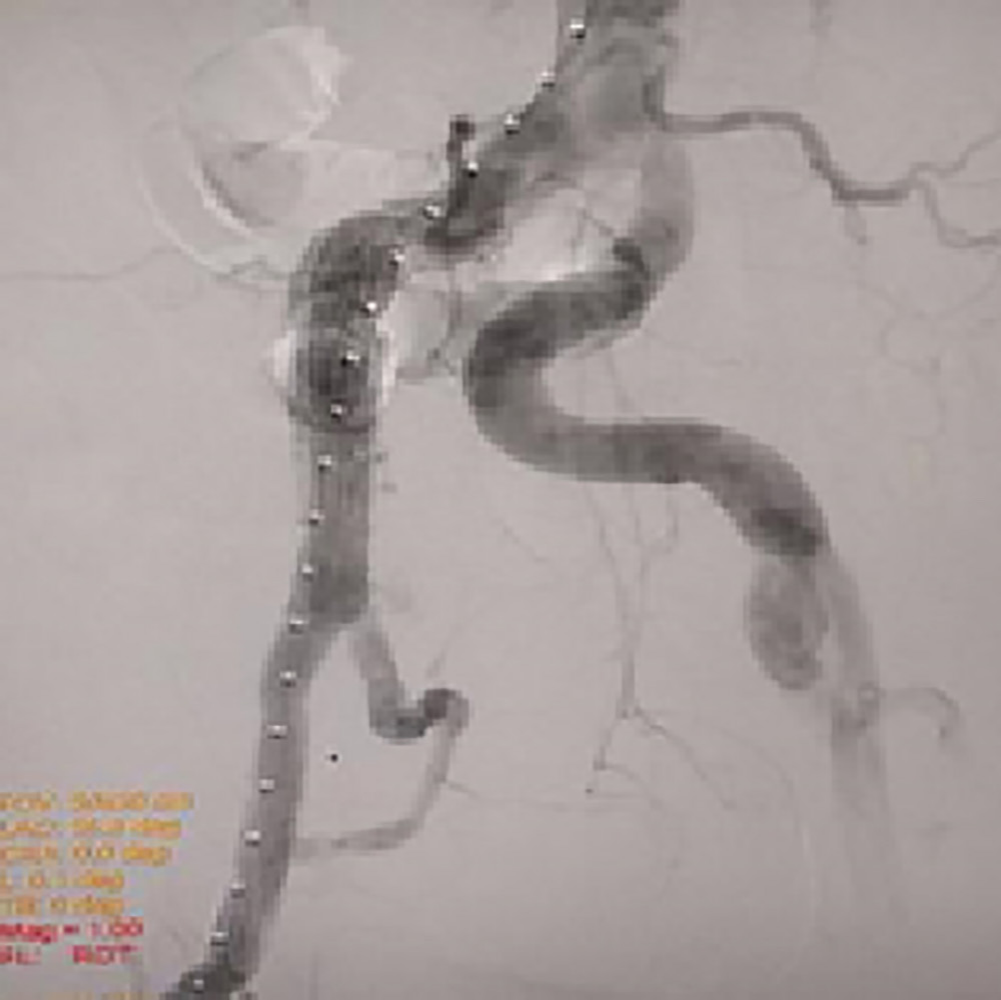
Arteriography at completion of the first procedure.

The right common femoral artery was dissected, followed initially by insertion of an 8 French introducer for angiography and road-mapping, and then an H&L-B One-Shot^®^ 20 F endoprosthesis deployment system was inserted, followed by placement of a ZBIS Zenith® bifurcated iliac endoprosthesis (12 mm diameter / 61 mm length - common iliac / 58 mm length - external iliac). Once the endoprosthesis had been positioned, a 12 F sheath was inserted via the left femoral artery over a pre-catheterized guidewire (providing access to the branch of the right internal iliac artery) and then a Lifestream^®^ balloon-expandable covered stent (8 mm diameter / 58 mm length) was released. The distal segment of the stent ended in the superior gluteal artery (which had a diameter of 5 mm). At the end of the procedure, angiographic results were satisfactory, with no endoleaks, and the right internal iliac artery had been successfully preserved (Figure 2). The Jotec E-iliac^®^ branched iliac endoprosthesis was not employed in this case, although it would have been an appropriate choice, since it is available with common iliac diameters of 14, 16, or 18 mm, making it more compatible with the case described here.

**Figure 3:**
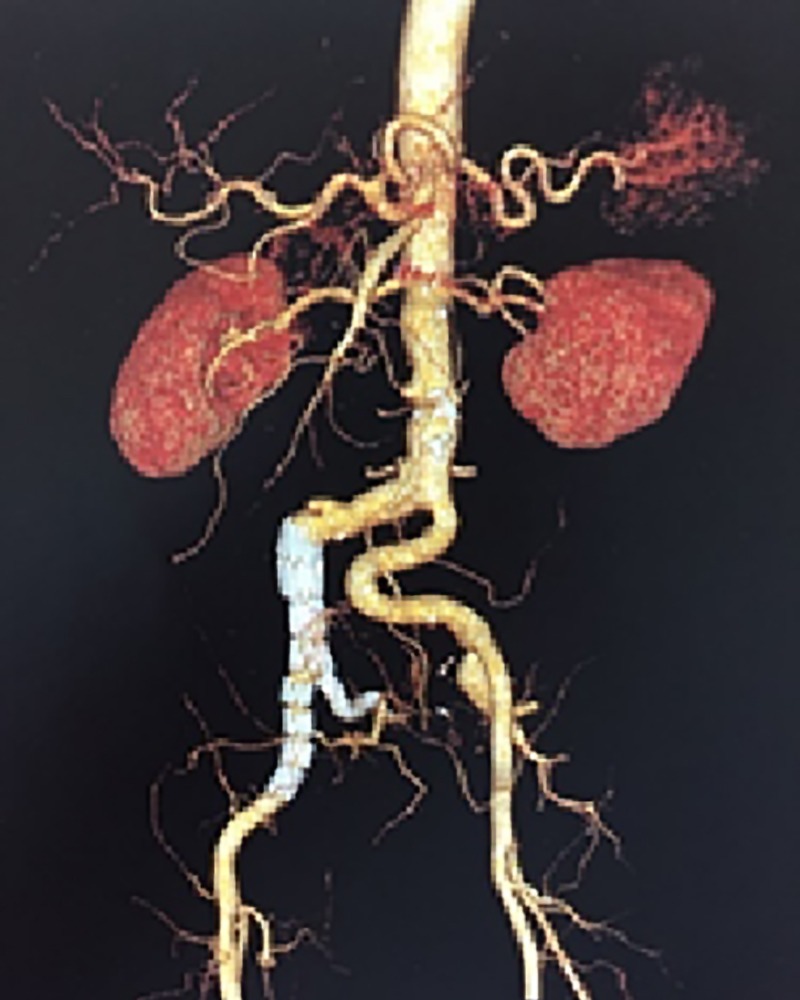
Angiotomography showing correctly implanted endoprosthesis.

Angiotomography was conducted again 1 month after the procedure, showing the endoprosthesis correctly implanted, with no areas of stenosis or leakage (Figure 3).

**Figure 4:**
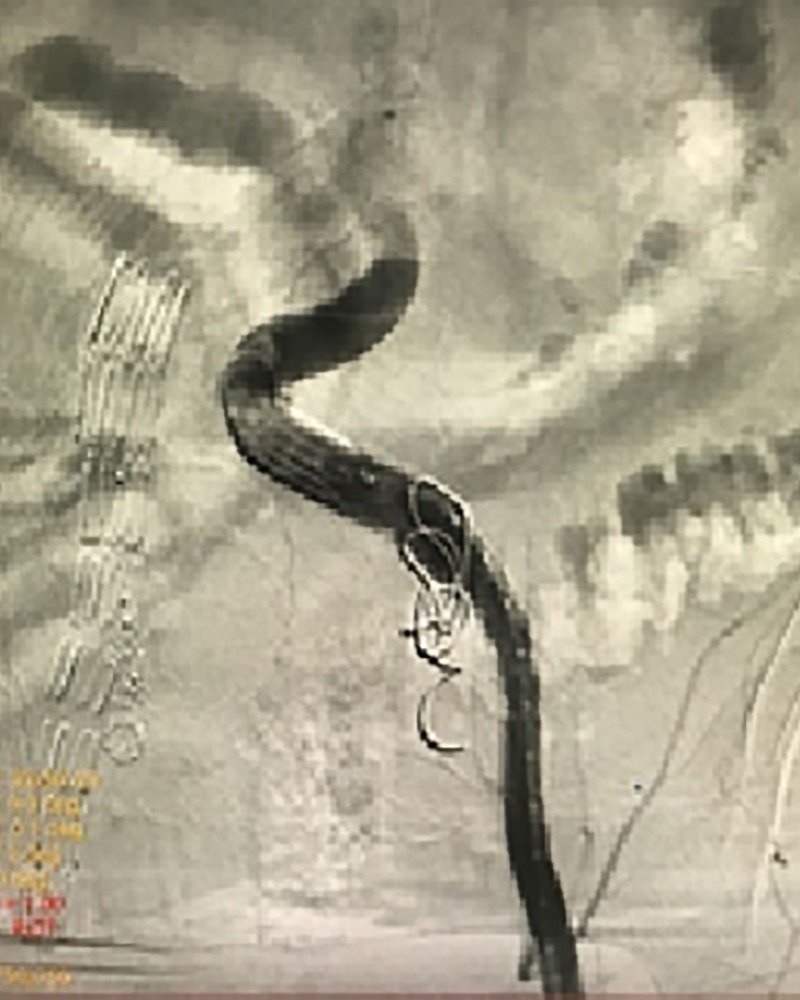
Arteriography at completion of the second procedure.

After 60 days had elapsed, the left internal iliac artery aneurysm was treated. This procedure comprised embolization of the aneurysm with two coils 14 mm in diameter by 14 cm in length (Nester^®^, COOK^®^) deployed via a Cobra 1 catheter after dissection of the left common femoral artery to insert a Z-Trak^®^ 14 F introduction system and release of a ZSLE Zenith^®^ iliac extension endoprosthesis (13 mm diameter / 90 mm length) to seal the ostium of the internal iliac, to prevent future filling and pressurization of the aneurysm. Angiography conducted at the end of the procedure showed the coils correctly implanted and the endoprosthesis patent and free from leakage (Figure 4).

It is important to point out that the only ZBIS endoprosthesis proximal body diameter available is 12 mm. The manufacturer recommends that the diameter of the common iliac artery adjacent to the branch should be, at least, 16 mm, enabling the branch and the device to open completely. This is why we used the ZBIS on the right side (where the common iliac diameter was 18 mm) and not on the left side (where the common iliac diameter was 14 mm). The sandwich technique is another option that was considered to maintain patency on the left side. However, because of unavailability of materials and a lack of familiarity with the technique, we preferred to perform embolization, since we had successfully maintained patency of the right internal iliac artery.

The patient was discharged on the day after the procedure, in good general condition, taking antiplatelet drugs. He recovered satisfactorily during the postoperative period, with no gastrointestinal abnormalities, gluteal claudication, or erectile dysfunction. At 6-month and 1-year follow-up assessments, he was asymptomatic and there was no evidence of endoleaks on angiotomography.

## DISCUSSION

Iliac artery aneurysms occur in 0.3-0.6% of the population in general.^1^ The common iliac artery is involved in 70-90% of cases (56% bilaterally), the internal iliac in 0-30% (50% bilaterally), and the external in 10%. There is a predominance among men (5:1) and the elderly.^2^^-^ 6 ^,^ 20 ^,^ 21

Aneurysms can be classified as congenital or acquired and as saccular or fusiform (which are more frequent and are associated with atherosclerotic disease). Etiologic factors include traumas, vasculitis, pregnancy, infections, connective tissue diseases, and iatrogeny.^2^^,^ 3 ^,^ 6 ^,^ 8 ^,^ 22

The majority of IAA are asymptomatic, making diagnosis difficult. They can manifest with pulsating masses, abdominal and/or lower back pain (acute, caused by expansion or rupture, or chronic, due to compression of nerves and visceral organs), with urinary symptoms (54%), gastrointestinal symptoms (constipation, tenesmus, pain on digital rectal examination, and enterorrhagia), and neurological symptoms, with thromboembolic phenomena caused by compression of the iliac-femoral venous system,^2^ or even with arteriovenous fistula, when an iliac aneurysm ruptures into an adjacent vein.^23^ However, the majority of diagnoses are made incidentally during laparotomies, necropsies, and imaging exams ordered to investigate other conditions.^3^ In cases that rupture, mortality rates are elevated, ranging from 33 to 50%.^7^^,^ 8 ^,^ 24 Elective therapeutic procedures, including endovascular techniques, have lower mortality rates, oscillating between 0 and 11%.^24^

Ultrasonography is frequently involved in incidental diagnosis of IAA, and is useful in diagnosis, screening, and follow-up of asymptomatic patients. Helical computed angiotomography is the gold standard, showing site, size, tortuosity, path, relationship with adjacent organs, signs of rupture, and retroperitoneal hemorrhage. Magnetic resonance angiography is used in cases for which iodinated contrast is contraindicated.^2^^,^ 3 ^,^ 8

Open surgical treatment of iliac aneurysms is challenging, because of the pelvic topography of these aneurysms and the risks caused by proximity to important adjacent structures.^2^

The preferred open treatment of aneurysms in the iliac area is proximal and distal ligature of the aneurysm and its tributaries. In the case of bilateral involvement, it is necessary to investigate inferior mesenteric artery patency, since if this artery is subject to significant occlusion or atheromatosis, there is a high risk of ischemia of pelvic organs or sexual dysfunction. Although ligature of aneurysms is an effective treatment, it is associated with a high risk of intraoperative bleeding, with mortality rates as high as 28%.^4^ The majority of authors recommend preserving at least one internal iliac artery, thereby avoiding complications such as ischemia of the colon, ischemia of the spinal cord with paraplegia, gluteal necrosis, gluteal claudication, and erectile dysfunction.^2^^,^ 5 The technique of excising and resecting these aneurysms is also associated with a high risk of bleeding and high rates of injuries to the ureter and adjacent structures, in addition to similar mortality (26.7%). Endoaneurysmorrhaphy is not recommended for bilateral iliac aneurysms, because of the high risk of gluteal necrosis, colitis, and paralysis.^4^

Endovascular treatment is associated with reduced surgical trauma, shorter length of hospital stay, and lower blood loss, with quicker postoperative recovery,^3^^,^ 8 and is the preferred treatment in these cases. The endovascular technique of embolization of the aneurysmal artery with coils is associated with complications such as gluteal claudication, in 12-55%, and erectile dysfunction, in 1-13% of the patients.^4^ With regard to preservation of the internal iliac artery, branched iliac endoprostheses^14^^-^ 19 have a success rate of 85-100% when modern devices are employed.^14^^,^ 25

According to the recommendations of the manufacturer of the endoprosthesis employed in the patient described here, for cases in which aneurysms involve the iliac segment, branched endoprostheses are only indicated when the patient has an external iliac artery landing site with a minimum length of 20 mm, an external iliac artery diameter that does not exceed 11 mm and is no less than 8 mm, and femoral artery calibers compatible with passage of the introducers, which have an external diameter of 7.7 mm.

The case presented here demonstrates an infrequent condition in the overall set of aneurysmal diseases (the internal iliac artery is involved in just 10-30% of cases in the 0.3-0.6% of the general population who have iliac aneurysms),^1^^-^ 6 treated with a bilateral endovascular approach. Knowledge of the many different techniques and devices, including their limitations, is of fundamental importance to adequate planning of endovascular techniques, even in uncommon cases, such as the one presented here.
