# Supplementary material for: Endovascular treatment of bilateral isolated aneurysm of the internal iliac artery
Source: J Vasc Bras. 2019 Apr 17;18:e20180115. doi: 10.1590/1677-5449.180115 (PMC6629456; doi:10.1590/1677-5449.180115)
Supplement: Translation

# Translation: Tratamento endovascular de aneurisma isolado bilateral de artéria ilíaca interna

**Authors:** Edwaldo Edner Joviliano, Daniela Vieira, Letícia da Silva Moreira, André Luís Foroni Casas

**Affiliations:** 1 Cirurgia Vascular, Universidade de São Paulo – USP, Ribeirão Preto, SP, Brasil.; 2 Cirurgia Vascular, Universidade de Franca – UNIFRAN, Franca, SP, Brasil.

**Keywords:** aneurisma ilíaco, procedimentos endovasculares, stents

## Abstract

Os aneurismas isolados das artérias ilíacas representam menos de 2% de todos os aneurismas abdominais. A artéria ilíaca interna é acometida em 10-30% dos casos. Na maioria dos casos os pacientes são assintomáticos, exceto se houver ruptura. Os aneurismas podem ser diagnosticados por ultrassom Doppler, ressonância magnética ou, preferencialmente, angiotomografia. As indicações cirúrgicas são expansão significativa, 3 ou mais cm de diâmetro ou casos sintomáticos. Apresentamos o caso de um paciente com achado ultrassonográfico incidental de aneurisma bilateral das artérias ilíacas internas, ambos com indicação cirúrgica. O paciente foi submetido a tratamento endovascular com sucesso, primeiramente com preservação da artéria ilíaca interna direita com endoprótese ramificada de ilíaca, seguida de embolização da artéria ilíaca interna esquerda. O conhecimento das diversas técnicas e dispositivos, assim como suas limitações, é fundamental para um adequado planejamento terapêutico endovascular, mesmo em casos pouco frequentes, como o aqui apresentado.

## INTRODUÇÃO

Os aneurismas de artéria ilíaca (AAI) representam menos de 2% de todos os aneurismas abdominais e ocorrem em 0,3-0,6% da população em geral^1^. A artéria ilíaca interna é acometida em 10-30% dos casos, sendo que em metade das vezes o aneurisma é bilateral^2^^-^ 6. A taxa de mortalidade é de 10% dos casos tratados com procedimentos eletivos abertos. Quando ocorre a ruptura do aneurisma, a mortalidade atinge 33-50%^7^^,^ 8. Alguns casos apresentam-se inicialmente dessa forma, dificultando o tratamento.

A maioria dos AAI é assintomática, dificultando o diagnóstico^2^, sendo que a maioria permanece assintomática até a ruptura ou o diagnóstico incidental^9^. A ruptura, que ocorre em 38-51% dos casos, caracteriza-se pela tríade de dor aguda e progressiva, hipotensão e massa pulsátil localizada em abdome inferior e regiões inguinais. Os AAI podem ocorrer no espaço retro ou intraperitoneal, comprimindo reto, ureter ou bexiga^8^. Recentemente, a maior disponibilidade e sensibilidade alcançadas pelas técnicas de imagem levaram a um aumento do diagnóstico precoce desses aneurismas^9^.

Exames de imagem não invasivos são frequentemente envolvidos no diagnóstico incidental, porém o exame padrão-ouro é a angiotomografia helicoidal^2^^,^ 3 ^,^ 8.

A história natural dos aneurismas de artéria ilíaca interna ainda não é clara, porém vários autores recomendam a correção desses aneurismas quando maiores do que 3 cm de diâmetro, pois o risco de ruptura nesses casos foi de 14-31%. O manejo clínico fica reservado para os pacientes com aneurismas menores do que 3 cm e assintomáticos. Ressalta-se, entretanto, que já houve relatos de ruptura em aneurismas com menos de 3 cm de diâmetro^4^. Dentre as abordagens técnicas possíveis, destacam-se a endovascular e a aberta. Atualmente, considera-se a cirurgia aberta apenas em casos com variações anatômicas, pois possui elevados índices de complicações e mortalidade (11 a 33%)^3^. Além disso, os aneurismas ilíacos internos apresentam peculiaridades que dificultam a abordagem aberta (localização profunda na pelve, relação íntima com as veias, proximidade com o ureter e difícil exposição dos ramos distais). O tratamento endovascular é atualmente o de escolha, já que é menos invasivo, diminui o risco de hemorragias intraoperatórias e de insuficiência renal e reduz o tempo de permanência intra-hospitalar^2^^,^ 10 ^,^ 11.

Apesar de haver inúmeras técnicas endovasculares disponíveis, a escolha do tratamento depende da anatomia da lesão e da experiência da equipe. Habitualmente recomenda-se exclusão do aneurisma, o que acarreta menos complicações e melhor resultado a longo prazo^2^^,^ 5 ^,^ 12 ^-^ 19.

### Parte I – Situação clínica

Homem, 69 anos de idade, com história prévia de dor em região hipogástrica e em flanco esquerdo. Durante a investigação do quadro álgico, foi solicitado um ultrassom de rins e vias urinárias. O exame não demonstrou anormalidades no sistema urinário, porém detectou aneurismas em ambas as artérias ilíacas internas. Como fator de risco, o paciente apresentava apenas hipertensão arterial sistêmica. Não havia histórico familiar de doença aneurismática.

Na ocasião, o paciente encontrava-se em bom estado geral. Negava impotência sexual ou queixas álgicas. Apresentava pressão arterial de 130/80 mmHg e frequência cardíaca de 70 batimentos por minuto. Exame pulmonar e cardíaco não revelou qualquer anormalidade. O abdome apresentava-se plano, flácido e indolor à palpação. Pulsos femorais, poplíteos, tibiais posteriores e dorsais dos pés apresentavam-se palpáveis, com índice tornozelo-braquial normal bilateralmente. O paciente utilizava losartana potássica 50 mg via oral uma vez ao dia.

A angiotomografia demonstrou aneurisma fusiforme da artéria ilíaca interna direita, com diâmetro máximo de 4,2 cm e extensão longitudinal de 5,9 cm, e aneurisma fusiforme da artéria ilíaca interna esquerda, com diâmetro máximo de 3,5 cm e extensão longitudinal de 4,7 cm (Figura 1). A aorta infrarrenal media 20 mm de diâmetro. A artéria ilíaca comum direita possuía 18 mm de diâmetro e a artéria ilíaca comum esquerda 14 mm. As artérias ilíacas externas possuíam 11 mm de diâmetro. O exame angiotomográfico dos membros inferiores encontrava-se normal.

**Figura 1:** Aneurismas fusiformes em artérias ilíacas internas.

Os exames laboratoriais demonstravam hemoglobina: 12,4 g/dL; hematócrito: 36%; plaquetas: 408.000/mm3; glicose: 85 mg/dL; sódio: 142 mEq/mL; potássio: 3,7 mEq/L; creatinina: 1,0 mg/dL; ureia: 40 mg/dL; tempo de tromboplastina parcialmente ativada (TTPA): 33,8 s; razão normalizada internacional (*international normalized ratio*, INR): 1,2 e atividade de protrombina de 62%.

Diante desse quadro, algumas opções terapêuticas foram aventadas:

1 - Endoprótese ramificada de ilíaca bilateral, com preservação de ambas as artérias ilíacas internas;
2 - Endoprótese ramificada de ilíaca unilateral, com preservação de uma artéria ilíaca interna e embolização da outra;
3 - Embolização de ambas as artérias ilíacas internas sequencialmente;
4 - Endoprótese aortoilíaca;
5 - Tratamento cirúrgico aberto, com ligadura dos aneurismas.

### Parte II – O que foi feito

Optou-se pelo tratamento endovascular, com a intenção de tratar primeiramente o maior aneurisma e preservar a artéria ilíaca interna, para posteriormente realizar o tratamento (embolização) do menor aneurisma.

Através de dissecção da artéria femoral comum direita e colocação inicial de introdutor de número 8 *French* (F) para angiografia e *road-mapping*, foi colocado o sistema de introdução da endoprótese H&L-B One-Shot^®^ 20 F e realizado o posicionamento de endoprótese bifurcada de ilíaca ZBIS Zenith^®^ (12 mm de diâmetro / 61 mm de comprimento - ilíaca comum / 58 mm de comprimento - ilíaca externa). Após o posicionamento da endoprótese, foi introduzida uma bainha 12 F via artéria femoral esquerda através do fio-guia pré-cateterizado (que dava acesso ao ramo da artéria ilíaca interna direita), sendo liberado um stent revestido expansível por balão da marca Lifestream^®^ (8 mm de diâmetro / 58 mm de comprimento). O segmento distal do stent terminou na artéria glútea superior (que possuía 5 mm de diâmetro). Ao final do procedimento, observou-se resultado angiográfico satisfatório, sem ocorrência de vazamentos (*endoleaks*) e com preservação da artéria ilíaca interna direita (Figura 2). A endoprótese ramificada de ilíaca Jotec E-iliac^®^ não foi utilizada neste caso, porém seria uma opção adequada, já que dispõe diâmetros da ilíaca comum de 14, 16 ou 18 mm, mais compatíveis com o caso em questão.

**Figura 2:** Arteriografia final do primeiro procedimento.

Foi realizada nova angiotomografia 1 mês após o procedimento, que demonstrou endoprótese adequadamente implantada, sem áreas de estenoses ou vazamentos (Figura 3).

**Figura 3:** Angiotomografia evidenciando endoprótese corretamente implantada.

Após 60 dias, foi realizada a abordagem do aneurisma da artéria ilíaca interna esquerda. O procedimento ocorreu com a realização de embolização do aneurisma com duas molas de 14 mm de diâmetro / 14 cm de comprimento (Nester^®^, COOK^®^) através de cateter Cobra 1 e de dissecção da artéria femoral comum esquerda, com posicionamento do sistema de introdução Z-Trak^®^ 14 F e implante de endoprótese extensão ilíaca ZSLE Zenith^®^ (13 mm de diâmetro / 90 mm de comprimento) para selamento do óstio da ilíaca interna, a fim de evitar enchimento e pressurização futura do aneurisma. Foi realizada angiografia ao final do procedimento, demonstrando molas adequadamente implantadas, endoprótese patente e ausência de vazamentos (Figura 4).

**Figura 4:** Arteriografia final do segundo procedimento.

É importante observar que o único diâmetro do corpo proximal disponível para a endoprótese ZBIS é de 12 mm. O fabricante recomenda que o diâmetro da artéria ilíaca comum adjacente à ramificação deve ser de, pelo menos, 16 mm. Isso permite que a ramificação e o dispositivo abram na totalidade. Por esse motivo, utilizamos a ZBIS no lado direito (onde a ilíaca comum possuía 18 mm de diâmetro) e não a utilizamos no lado esquerdo (onde a ilíaca comum possuía 14 mm de diâmetro). A técnica do *sandwich* foi uma alternativa aventada para manter perviedade do lado esquerdo. Porém pela indisponibilidade de materiais e da familiaridade com a técnica, preferimos realizar a embolização, já que havíamos conseguido manter a perviedade da artéria ilíaca interna direita.

O paciente recebeu alta no dia seguinte ao procedimento em bom estado geral, em uso de antiagregante plaquetário. Evoluiu satisfatoriamente no pós-operatório, com ausência de anormalidades gastrointestinais, claudicação glútea ou disfunção erétil. Nas avaliações de 6 meses e 1 ano após o procedimento, encontrava-se assintomático e sem evidência de vazamentos (*endoleaks*) nas angiotomografias de controle.

## DISCUSSÃO

Os aneurismas de artéria ilíaca ocorrem em 0,3-0,6% da população em geral^1^. A artéria ilíaca comum é acometida em 70-90% das vezes (56% bilateral), a interna em 10-30% (50% bilateral), e a externa em 10%. Há predomínio em homens (5:1) e em idosos^2^^-^ 6 ^,^ 20 ^,^ 21.

Os aneurismas podem ser classificados em congênitos ou adquiridos e em saculares ou fusiformes (que são os mais frequentes e estão associados à doença aterosclerótica). Alguns fatores etiológicos são traumas, vasculites, gravidez, infecções, doenças do colágeno e iatrogenia^2^^,^ 3 ^,^ 6 ^,^ 8 ^,^ 22.

A maioria dos AAI é assintomática, dificultando o diagnóstico. Podem manifestar-se com massa pulsátil, dor abdominal e/ou lombossacra (aguda, por expansão ou ruptura ou crônica, por compressão de nervos e vísceras), com sintomas urinários (54%), gastrointestinais (constipação, tenesmo, dor ao toque retal e enterorragia) e neurológicos, com fenômenos tromboembólicos por compressão do sistema venoso ilíacofemoral^2^ ou até mesmo com fístula arteriovenosa, quando há ruptura de um aneurisma ilíaco para o interior de uma veia adjacente^23^. No entanto, a maior parte do diagnóstico ocorre incidentalmente durante laparotomias, necropsias e em exames de imagem para investigação de outras condições^3^. Em casos de ruptura, as taxas de mortalidade são elevadas, variando entre 33-50%^7^^,^ 8 ^,^ 24. Os procedimentos terapêuticos eletivos, incluindo técnicas endovasculares, apresentam menores taxas de mortalidade, oscilando entre 0 a 11%^24^.

A ultrassonografia é frequentemente envolvida no diagnóstico incidental dos AAI, sendo útil no diagnóstico, na triagem e no seguimento dos pacientes assintomáticos. A angiotomografia helicoidal é o padrão-ouro, evidenciando localização, tamanho, tortuosidade, trajeto, relação com órgãos adjacentes, sinais de ruptura e hemorragia retroperitoneal. Emprega-se a angiorressonância em casos de contraindicação ao contraste iodado^2^^,^ 3 ^,^ 8.

O tratamento cirúrgico aberto dos aneurismas ilíacos é desafiador, devido à topografia pélvica desses aneurismas e aos riscos oferecidos pela proximidade com importantes estruturas adjacentes^2^.

O tratamento aberto preferencial dos aneurismas do território ilíaco é a ligadura proximal e distal do aneurisma e de suas tributárias. No caso de envolvimento bilateral, é necessário verificar a perviedade da artéria mesentérica inferior, pois caso esta apresente oclusão ou ateromatose significativa, existe um alto risco de isquemia de órgãos pélvicos ou de impotência sexual. Apesar de proporcionar um tratamento efetivo, a ligadura dos aneurismas apresenta alto risco de sangramento intraoperatório, com taxas de mortalidade que chegam a 28%^4^. A maioria dos autores preconiza preservar pelo menos uma artéria ilíaca interna, evitando complicações como isquemia cólica, isquemia medular com paraplegia, necrose glútea, claudicação glútea e disfunção erétil^2^^,^ 5. A técnica de excisão e ressecção desses aneurismas também demonstrou alto risco de sangramento e altos índices de lesão de ureter e de estruturas adjacentes, além de apresentar mortalidade semelhante (26,7%). A endoaneurismorrafia não é recomendada em aneurismas ilíacos bilaterais, pelo alto risco de necrose glútea, colite e paralisia^4^.

O tratamento endovascular está associado a uma diminuição do trauma cirúrgico, do tempo de internação e da perda sanguínea, com recuperação pós-operatória mais rápida^3^^,^ 8, sendo o tratamento preferido nestes casos. A modalidade endovascular por embolização da artéria aneurismática através de molas está associada a complicações como claudicação glútea em 12-55% e disfunção erétil em 1-13% dos pacientes^4^. Em se tratando de preservação da artéria ilíaca interna, a endoprótese ramificada de ilíaca^14^^-^ 19 tem taxa de sucesso de 85-100% quando foram utilizados dispositivos modernos^14^^,^ 25.

Segundo recomendação do fabricante da endoprótese utilizada no paciente aqui descrito, nos casos de aneurismas envolvendo o segmento ilíaco, as endopróteses ramificadas possuem indicação apenas quando o paciente possuir segmento de fixação de artéria ilíaca externa com comprimento mínimo de 20 mm, diâmetro da artéria ilíaca externa não superior a 11 mm e não inferior a 8 mm, além de calibre das artérias femorais compatível com a navegação dos introdutores, que possuem diâmetro externo de 7,7 mm.

O caso apresentado demonstrou uma condição pouco frequente no universo das doenças aneurismáticas (a artéria ilíaca interna é envolvida em apenas 10-30% dos casos dentre 0,3-0,6% da população geral que possui aneurismas no território ilíaco^1^^-^ 6) tratados com abordagem endovascular bilateral. O conhecimento das diversas técnicas e dispositivos, assim como suas limitações, é fundamental para um adequado planejamento das técnicas endovasculares, mesmo em casos pouco frequentes, como o aqui apresentado.
